# Heart failure care and outcomes in a Tanzanian emergency department: A prospective observational study

**DOI:** 10.1371/journal.pone.0254609

**Published:** 2021-07-13

**Authors:** Sainikitha Prattipati, Francis M. Sakita, Godfrey L. Kweka, Tumsifu G. Tarimo, Timothy Peterson, Blandina T. Mmbaga, Nathan M. Thielman, Alexander T. Limkakeng, Gerald S. Bloomfield, Julian T. Hertz

**Affiliations:** 1 Duke Global Health Institute, Durham, North Carolina, United States of America; 2 Kilimanjaro Christian Medical Centre, Moshi, Tanzania; 3 Kilimanjaro Christian Medical Centre University College, Moshi, Tanzania; 4 Division of Emergency Medicine, Duke University School of Medicine, Durham, North Carolina, United States of America; 5 Kilimanjaro Christian Research Institute, Moshi, Tanzania; 6 Kilimanjaro Christian Medical University College, Moshi, Tanzania; 7 Department of Internal Medicine, Duke University School of Medicine, Durham, North Carolina, United States of America; 8 Division of Cardiology, Duke University School of Medicine, Durham, North Carolina, United States of America; 9 Duke Clinical Research Institute, Durham, North Carolina, United States of America; Maastricht University Medical Center, NETHERLANDS

## Abstract

**Background:**

The burden of heart failure is growing in sub-Saharan Africa, but there is a dearth of data characterizing care and outcomes of heart failure patients in the region, particularly in emergency department settings.

**Methods:**

In a prospective observational study, adult patients presenting with shortness of breath or chest pain to an emergency department in northern Tanzania were consecutively enrolled. Participants with a physician-documented clinical diagnosis of heart failure were included in the present analysis. Standardized questionnaires regarding medical history and medication use were administered at enrollment, and treatments given in the emergency department were recorded. Thirty days after enrollment, a follow-up questionnaire was administered to assess mortality and medication use. Multivariate logistic regression was performed to identify baseline predictors of thirty-day mortality.

**Results:**

Of 1020 enrolled participants enrolled from August 2018 through October 2019, 267 patients (26.2%) were diagnosed with heart failure. Of these, 139 (52.1%) reported a prior history of heart failure, 168 (62.9%) had self-reported history of hypertension, and 186 (69.7%) had NYHA Class III or IV heart failure. At baseline, 40 (15.0%) reported taking a diuretic and 67 (25.1%) reported taking any antihypertensive. Thirty days following presentation, 63 (25.4%) participants diagnosed with heart failure had died. Of 185 surviving participants, 16 (8.6%) reported taking a diuretic, 24 (13.0%) reported taking an antihypertensive, and 26 (14.1%) were rehospitalized. Multivariate predictors of thirty-day mortality included self-reported hypertension (OR = 0.42, 95% CI: 0.21–0.86], *p* = 0.017) and symptomatic leg swelling at presentation (OR = 2.69, 95% CI: 1.35–5.56, *p* = 0.006).

**Conclusion:**

In a northern Tanzanian emergency department, heart failure is a common clinical diagnosis, but uptake of evidence-based outpatient therapies is poor and thirty-day mortality is high. Interventions are needed to improve care and outcomes for heart failure patients in the emergency department setting.

## Introduction

The burden of cardiovascular disease in sub-Saharan Africa (SSA) has increased as it proceeds through the epidemiological shift from infectious diseases to chronic non-communicable diseases [1]. Heart failure (HF) is a terminal complication of many cardiovascular diseases, and is responsible for a growing number of hospitalizations in SSA [2–6]. With the rapid increase in the prevalence of HF risk factors in SSA, the burden of HF is expected to grow in future years [1, 7].

Chronic diseases such as HF require robust health systems that can provide integrated, long-term, continuous care [8, 9]. Weak health systems, limited access to primary care, and an insufficient workforce hamper efforts to curb the growing burden of HF in SSA [10, 11]. Moreover, limited access to diagnostic technology such as echocardiography and shortages of essential HF medications make it difficult to recognize and treat HF in many healthcare facilities in SSA [12–14]. These challenges have resulted in sub-optimal HF care and outcomes in SSA. HF patients in SSA tend to be younger at disease onset, generally present later in their disease progression, and experience worse outcomes than patients in other regions of the world [15, 16]. Indeed, recent studies found that patients in SSA with HF suffer from the highest in-hospital mortality, 12-month mortality, and readmission rates in the world [17, 18].

Little is known about HF outcomes among patients presenting for acute care in SSA. Much of the existing data regarding HF in SSA comes from studies of hospitalized patients at advanced specialty cardiology hospitals [19, 20]. Since the majority of patients in SSA do not have access to such specialty hospitals, more data are needed to describe HF care in typical SSA settings [21, 22]. Furthermore, in the absence of strong primary care systems, patients with HF in SSA increasingly seek care in acute care settings like emergency departments (EDs), but studies of ED HF management in SSA are scarce. To address this knowledge gap, this study aimed to describe the prevalence, management, and outcomes of HF at an ED in northern Tanzania.

## Methods

### Ethics statement

Written informed consent was obtained from all participants prior to enrollment. This study received approval from the Kilimanjaro Christian Medical Centre Institutional Review Board, the National Health Research Ethics Committee of the Tanzania National Institute for Medical Research, and the Duke Health Institutional Review Board.

### Setting

This study was conducted in the ED at Kilimanjaro Christian Medical Centre (KCMC), a tertiary care center in northern Tanzania. KCMC serves a catchment population of over 15 million people, and the KCMC ED sees approximately 30,000 patients per year. The community prevalence of hypertension and diabetes among adults in northern Tanzania in 2014 was 28% and 6%, respectively [23, 24]. KCMC ED physicians have undergone residency training in either emergency medicine or internal medicine. At the time of this study, KCMC had echocardiography capacity but did not have any formally trained cardiologists on staff, and did not have access to BNP testing.

### Participant selection

This study was conducted from 20 August 2018 through 12 October 2019. The data collection protocol has been described in detail elsewhere [25]. Trained research assistants screened all adult (age ≥ 18 years) patients presenting to the KCMC ED from 8AM until 11PM daily. Any adult with a primary or secondary complaint of shortness of breath or chest pain was eligible for enrollment. Exclusion criteria were self-reported fever, chest pain secondary to trauma, and inability to provide informed consent. Participants were enrolled consecutively.

### Study procedures

After obtaining informed consent, trained research assistants administered a detailed survey collecting information about sociodemographics and medical history based on the World Health Organization (WHO) STEPS survey [26]. The patient survey is provided as S1 File. Participants were also asked to provide information about the current symptoms that prompted them to seek care in the ED. Height, weight, and blood pressure were measured and recorded for each participant. Research assistants did not perform physical examinations of participants. Each participant’s treatments in the ED was recorded and clinical diagnoses were collected directly from the patient’s medical record at the conclusion of their ED visit. Research assistants did not have access to results of laboratory of radiology investigations when obtained by the ED physician. Thirty days following enrollment, participants were contacted via telephone to administer a follow-up survey to assess mortality, medication use, and symptom status. Participants were also asked to identify their primary ED diagnosis in their own words. If a participant was unreachable by telephone, a research assistant visited the participant’s home to administer the follow-up survey. If a participant was deceased, the survey was administered to a relative.

### Heart failure definition

HF was defined by a documented diagnosis of heart failure by the ED physician. Any participant with a physician-documented diagnosis of heart failure for their ED visit was included in the present analysis. ED physicians independently determined patient diagnoses based on all available data as per routine care; no standardized criteria were used for diagnosis. Etiology of heart failure was determined by the ED physician’s documented etiology for heart failure, as determined by his or her independent clinical judgment. If no etiology of heart failure was documented, the etiology was classified as “unspecified.”

### Other study definitions

Body mass index (BMI) was calculated directly from measured weight and height. Mean arterial pressure at presentation was calculated by adding one-third of the measured systolic blood pressure to two-thirds of the measure diastolic blood pressure. Pre-existing comorbidities such as hypertension, diabetes, and previously diagnosed HF were defined by patient self-report. Tobacco and alcohol use, medication use before and after the ED visit, and rehospitalization within thirty days of presentation were assessed by patient self-report. Sedentary lifestyle was defined as less than 150 minutes of moderately vigorous physical exercise per week, as per WHO guidelines [26]. Heart failure severity was determined by participant self-reported symptom severity, according to the New York Heart Association (NYHA) functional classification system [27]. An adjudication committee consisting of physicians from Tanzania and the United States, fluent in both Swahili and English, reviewed patient-reported diagnosis to determine whether or not the participant was able to accurately identify their diagnosis of HF. For example, participants who reported their diagnosis as “heart not pumping well” were categorized as able to identify their diagnosis, whereas those who reported not knowing their diagnosis or who reported a diagnosis not connected to HF (such as “malaria”) were categorized as being unable to identify their HF diagnosis. Disagreements among committee members were resolved by majority vote after discussion.

### Statistical analyses

All participants with a physician-documented clinical diagnosis of HF were included in the present analysis. Descriptive statistics, including medians (interquartile ranges) and frequencies were used to summarize baseline participant characteristics and ED treatments. Univariate associations between baseline participant characteristics and thirty-day mortality were assessed with Welch’s t-test for continuous variables and Pearson’s chi-squared for categorical variables. Fisher’s exact test was used for categorical variables with an expected cell count less than five. Multivariate logistic regression was then performed to identify predictors of thirty-day mortality among participants with HF. Any variable demonstrating potential association with thirty-day mortality (*p* < 0.10 in univariate analysis) was included in the model; sex and age were also forced into the model. Participants who were lost to follow-up were excluded from both univariate and multivariate mortality analyses. All statistical analyses were performed in the R Suite.

## Results

Of 1020 enrolled participants presenting to KCMC ED with shortness of breath or chest pain, 267 patients (26.2%) were clinically diagnosed with HF. The median (IQR) age of patients diagnosed with HF was 65 (51–78) years and 150 (56.2%) were female (**Table 1**). Approximately half the participants disclosed a prior history of HF (*n* = 139, 52.1%) prior to presentation. The most common presenting symptoms among participants were shortness of breath (*n* = 249, 93.3%), chest pain (*n* = 191, 71.5%), and leg swelling (*n* = 139, 52.1%).

**Table 1:** Characteristics of adults presenting to the KCMC emergency department and diagnosed with heart failure, 2018–2019 (n = 267).

Characteristic	n	(%)
Age, median (IQR), years	65 (51–78)	
Females	150	56.2
*Level of education*		
None	31	11.6
Primary	181	67.8
Secondary	33	12.4
Post-secondary	22	8.2
BMI, median (IQR), kg/m2	24.2 (20.8–28.7)	
*Self-reported co-morbidities*		
Hypertension	168	62.9
Diabetes	40	15.0
Chronic kidney disease	28	10.5
Hyperlipidemia	23	8.6
Ischemic heart disease	6	2.2
Known history of heart failure	139	52.1
Current alcohol use	72	27.0
Current tobacco use	17	6.4
Daily vegetable & fruit consumption	17	6.4
Sedentary lifestyle	153	57.3
*Medication use at baseline*		
Diuretic	40	15.0
Furosemide/lasix	37	13.9
HCTZ/thiazide	3	1.1
Spironolactone	2	0.7
Antihypertensive	67	25.1
Beta-blockers	9	3.4
Calcium channel blockers	13	4.9
ACE-inhibitors	10	3.7
Angiotensin receptor blockers	14	5.2
Nitrite	2	0.7
Other	35	13.1
Antiplatelet	17	6.4
*Presenting symptoms*		
Shortness of breath	249	93.3
Chest pain	191	71.5
Leg swelling	139	52.1
Palpitations	88	33.0
Light-headedness/syncope	21	7.9
Duration of symptoms prior to presentation, median (IQR), days	4 (3–14)	
Mean arterial pressure at presentation, median (IQR), mmHg	100.7 (84.7–115.8)	
Heart rate, median (IQR), beats per minute	93 (78–109)	
*NYHA Heart Failure Classification*		
Class I	47	17.6
Class II	34	12.7
Class III	99	37.1
Class IV	87	32.6

The majority of patients diagnosed with HF in the ED were hospitalized (n = 212, 79.4%; **Table 2**), for a median duration of a week.

**Table 2:** Emergency department care for adult patients diagnosed with heart failure, northern Tanzania, 2018–2019, (n = 267).

	n	(%)
*Treatments*		
Furosemide	66	24.7
Supplemental oxygen	50	18.7
Anti-hypertensive	20	7.5
Hospitalized	212	79.4
Duration of hospitalization if hospitalized, days, median (IQR)	7 (4–10)	
*Physician-documented heart failure etiology*		
Hypertensive heart disease	74	27.7
Ischemic heart disease	7	2.6
Valvular disease	2	0.7
Cardiomyopathy	5	1.9
Unspecified	179	67.0

**Fig 1:**
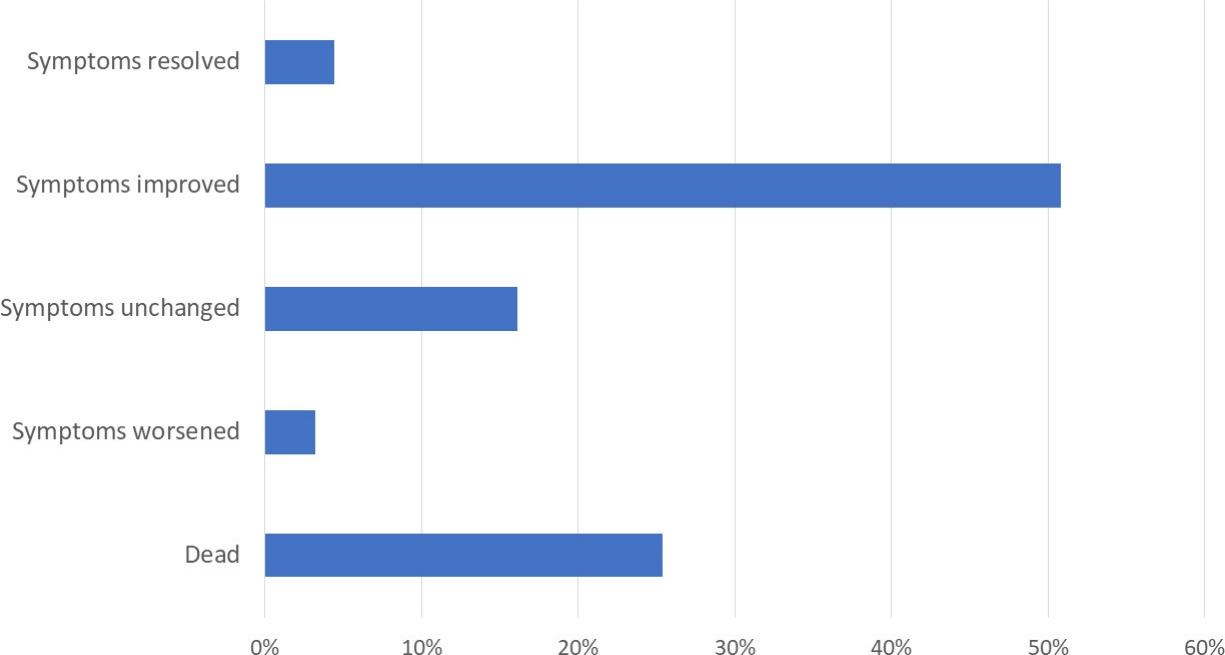
Thirty-day outcomes among patients diagnosed with heart failure in a Tanzanian emergency department, 2018–2019 (n = 248).

Thirty-day follow-up was completed for 248 (92.9%) participants diagnosed with HF; 19 (7.1%) participants were lost to follow-up. Of participants completing 30-day follow-up, 63 (25.4%) died within 30 days of hospital presentation. Thirty-day outcomes are summarized in **Fig 1**. Of the 185 participants surviving to thirty days, the majority reported their heart failure symptoms had improved (*n* = 126, 68.1%; **Table 3**). Of surviving participants, 26 (14.1%) were hospitalized within thirty days. Of surviving participants, 107 (57.8%) were able to identify their diagnosis as heart failure. At 30 days, 16 (8.6%) surviving participants reported taking a diuretic, and 24 (13.0%) reported taking any antihypertensive.

**Table 3:** Outcomes among patients surviving thirty days following hospital diagnosis of heart failure, northern Tanzania, 2018–2019 (n = 185)^a^.

	n	(%)
*Heart failure symptom status*		
Resolved	11	5.9
Improved	126	68.1
Unchanged	40	21.6
Worsened	8	4.3
Re-Hospitalized after discharge	26	14.1
Initial admission and subsequent re-hospitalization	19	9.7
Initial discharge and subsequent hospitalization	7	3.8
Received thirty-day follow-up appointment	57	30.8
*Medication use*		
Diuretic	16	8.6
Furosemide	15	8.1
HCTZ/thiazide	1	0.5
Antihypertensive	24	13.0
Beta-blockers	6	3.2
Calcium channel blockers	5	2.7
ACE inhibitors	6	3.2
Other	8	4.3
Antiplatelet	8	4.3
Able to identify heart failure as their diagnosis	107	57.8

^a^ excludes 63 patients who died within thirty days and 19 patients lost to follow-up

On univariate analysis, thirty-day mortality was associated with younger age (mean = 58.5 vs 65.0 years, *p* = 0.025; **Table 4**), lower mean arterial pressure at initial ED presentation (mean = 95.5 mmHg vs 103.3 mmHg, *p* = 0.023), current tobacco use (11.1% vs 4.9%, *OR* = 3.23, 95% CI = [0.98, 10.43], *p* = 0.036), leg swelling at presentation (74.6% vs 45.9%, OR = 3.42, 95% CI = [1.84, 6.65], *p* < 0.001), and a NYHA classification of Grade III or Grade IV (79.4% vs 64.9%, OR = 2.06, 95% CI = [1.07, 4.23], *p* = 0.032). Compared to patients who survived, patients who died within thirty days were also less likely to self-report a history of hypertension (41.3% vs 70.3%, OR 0.30, 95% CI = [0.16, 0.53], *p* < 0.001), less likely to report a history of hyperlipidemia (1.6% vs 9.7%, OR = 0.17, 95% CI = [0.01, 0.85], *p* = 0.036), and less likely to present with palpitations (22.2% vs. 36.8%, OR = 0.50, 95% CI = [0.25, 0.95], *p* < 0.034).

**Table 4:** Associations between baseline patient characteristics and thirty-day mortality following hospital diagnosis of heart failure, northern Tanzania, 2018–2019 (n = 248).

Baseline participant characteristic	Patients alive at 30 days (N = 185) n (%)	Patients dead at 30 days (N = 63) n (%)	OR (95% CI)	*p*^a^
Male sex	80 (43.2)	32 (50.8)	1.35 [0.76, 2.41]	0.298
No secondary education	145 (78.4)	52 (82.5)	1.29 [0.63, 2.83]	0.480
Self-reported history of hypertension	130 (70.3)	26 (41.3)	0.30 [0.16, 0.53]	< 0.001*
Self-reported history of diabetes	29 (15.7)	7 (11.1)	0.68 [0.26, 1.58]	0.374
Self-reported history of hyperlipidemia	18 (9.7)	1 (1.6)	0.17 [0.01, 0.85]	0.051^b^
Self-reported history of chronic kidney disease	19 (10.3)	8 (12.7)	1.28 [0.50, 3.02]	0.593
Known history of heart failure	100 (54.1)	31 (49.2)	0.82 [0.46, 1.47]	0.506
Current alcohol use	47 (25.4)	21 (33.3)	1.47 [0.78, 2.72]	0.223
Current tobacco use	9 (4.9)	7 (11.1)	2.44 [0.82, 6.97]	0.081
Daily vegetable and fruit consumption	12 (6.5)	2 (3.2)	0.50 [0.07, 1.94]	0.325
Sedentary lifestyle	105 (56.8)	41 (65.1)	1.41 [0.78, 2.60]	0.246
Diuretic use at baseline	29 (15.7)	6 (9.5)	0.58 [0.20, 1.39]	0.226
Antihypertensive use at baseline	43 (23.2)	15 (23.8)	1.04 [0.51, 2.01]	0.927
Antiplatelet use at baseline	15 (8.1)	2 (3.2)	0.40 [0.06, 1.48]	0.181
Presented with shortness of breath	171 (92.4)	60 (95.3)	1.57 [0.49, 7.33]	0.447
Presented with leg swelling	87 (47.0)	48 (76.2)	3.42 [1.84, 6.65]	< 0.001*
Presented with palpitations	68 (36.8)	14 (22.2)	0.50 [0.25, 0.95]	0.034*
Presented with chest pain	135 (73.0)	45 (71.4)	0.92 [0.49, 1.78]	0.812
Presented with light-headedness/syncope	15 (8.1)	4 (6.3)	0.79 [0.21, 2.31]	0.650
NYHA Classification III/IV	120 (64.9)	50 (79.4)	2.06 [1.07, 4.23]	0.032*
	**Patients alive at 30 days (N = 185), mean (sd)**	**Patients dead at 30 days (N = 63), mean (sd)**		***p***^c^
Age, years	65.0 (17.4)	58.5 (20.6)		0.025*
BMI, kg/m^2^	26.1 (6.7)	24.8 (10.5)		0.347
Mean arterial blood pressure at presentation, mmHg	103.3 (21.1)	95.5 (23.9)		0.023*

^a^ Pearson’s chi-squared test unless otherwise noted.

^b^ Fischer’s exact test

^c^ Welch’s t-test

*statistically significant of *p* < 0.05

On multivariate analysis, self-reported hypertension (OR = 0.42, 95% CI = [0.21, 0.86], *p* = 0.017) and leg swelling at presentation (OR = 2.69, 95% CI = [1.35, 5.56], *p* = 0.006) were statistically significant predictors of thirty-day mortality (**Table 5**).

**Table 5:** Multivariate predictors of thirty-day mortality following hospital diagnosis of heart failure, northern Tanzania (n = 248).

	OR (95% CI)	p-value
Male sex	1.08 [0.55, 2.09]	0.825
Age, years	0.99 [0.97, 1.01]	0.228
Self-reported Hypertension	0.42 [0.21, 0.86]	0.017*
Self-reported Hyperlipidemia	0.27 [0.01, 1.47]	0.221
Current tobacco use	2.34 [0.66, 8.36]	0.185
Presented with leg swelling	2.69 [1.35, 5.56]	0.006*
Presented with palpitations	0.66 [0.31, 1.34]	0.259
Mean arterial pressure, mmHg	1.00 [0.98, 1.01]	0.647
NYHA Classification III/IV	1.96 [0.94, 4.30]	0.079

*statistically significant of p < 0.05

## Discussion

This study is one of the first ED-based prospective studies of patients diagnosed with HF in SSA. We found that HF diagnosis was common among ED patients presenting with chest pain or shortness of breath, but many patients diagnosed with HF were unaware of their diagnosis and were not receiving guideline-recommended therapies. Moreover, thirty-day outcomes following hospital presentation were poor, with a quarter of HF diagnosis patients dying within 30 days of ED diagnosis and few surviving patients using evidence-based therapies.

HF was diagnosed in 26% of patients presenting to the ED with shortness of breath or chest pain, consistent with previous work finding HF to be a leading diagnosis among ED patients with these symptoms in SSA [5, 6, 28]. Importantly, only half the ED patients diagnosed with HF in Tanzania had a known prior diagnosis of HF, whereas the vast majority of HF patients presenting to EDs in high-income settings have a known prior history of HF [29–31]. This suggests that the ED may be a useful location for screening, diagnosis, and patient education of HF in Tanzania and elsewhere in SSA. Furthermore, diuretics and antihypertensives such as ACE inhibitors and beta blockers are guideline-recommended therapies to prevent HF exacerbations and treat its risk factors [13], but few HF patients in our setting reported taking such medications before or after their ED visit. For example, very few participants diagnosed with HF reported using diuretics or anti-hypertensive medications either before or after their ED visit. The reasons for low uptake of evidence-based HF medications are likely multifactorial, including a limited primary care system, fragmented systems of care leading to episodic healthcare encounters and short-term medication use, a dearth of specialist providers, cost of medications, inadequate patient education, and medication side effects, among others. Diuretics and anti-hypertensives are generally readily available in northern Tanzania, so medication shortages are likely not responsible for the observed low uptake of HF therapies in our study population. Further research is needed to understand the reasons for these findings.

Outcomes for patients diagnosed with HF were poor in northern Tanzania, with a 30-day mortality rate of 25%, similar to recent studies from elsewhere in SSA [17, 18, 32]. The mortality rates observed in SSA are much higher than what has been reported outside of SSA [33–35]: in the United States, for example, the 30-day mortality rate following ED presentation for HF is between 4 and 12 percent [36]. The higher mortality rate in Tanzania might be partly due to the low uptake of recommended HF therapies both before and following the ED visit.

In multivariate analysis, we found self-reported hypertension to negatively predict, and leg swelling to positively predict 30-day mortality among patients diagnosed with HF in northern Tanzania. With only about a quarter of the individuals with hypertension aware of their diagnosis in SSA [37, 38], patient self-report of hypertension is likely an indicator of engagement with the healthcare system and taking appropriate preventative therapies, which explains why self-reported hypertension appears to be protective against mortality among HF patients in our setting. Leg swelling is a marker of volume overload and is associated with severe symptoms and poor outcomes in HF, which may explain why leg swelling is a positive predictor of mortality in HF patients in Tanzania and elsewhere [39, 40]. Other studies have found age and NYHA class to be predictors of mortality in HF patients in SSA, although they were not predictive of mortality in our cohort [41–43]. Age and NYHA may not have been significant predictors of mortality in our study due to our relatively small sample size, our relatively short follow-up period examining thirty-day mortality, and the relatively larger impact of other factors such as engagement with the healthcare system in our setting. A longer study with more participants and longer follow-up would likely have allowed for a more robust assessment of predictors of mortality.

### Limitations

This study had several limitations. First, the study only included patients who survived to presentation at a referral hospital, so patients from more isolated, rural settings were likely underrepresented. Second, given the limited access to echocardiography in our setting, study inclusion was determined by an ED physician diagnosis of HF without complementary information or objective data such as echocardiographic findings. Although an imperfect diagnostic measure, multiple recent studies have shown that clinical diagnoses of HF are generally accurate even without access to echocardiography [44–47]. Nonetheless, in the case that any clinical diagnoses in the study were inaccurate, patients without HF may have been included in our cohort and patients with true HF may have been inappropriately excluded. Similarly, we relied on physician documentation to determine etiology of heart failure, but the accuracy of these physician determinations is unknown. Third, we lost a small number of participants to follow-up, which may have affected our analysis of predictors of mortality. Finally, we relied on patient report to determine medication use, which may have led to an under- or over-estimation of usage rates of guideline-recommended therapies.

## Conclusions

In conclusion, HF is a common clinical diagnosis in a Tanzanian ED, but patients diagnosed with HF experience high thirty-day mortality, inadequate follow-up, and high rates of rehospitalization. Moreover, many patients were not aware of their diagnosis and the majority were not taking guideline-recommended therapies either before or after their ED visit. The ED may be an important setting to screen for HF, initiate therapies, and education patients. Further study is needed to improve care and outcomes for HF patients in northern Tanzania.

## Supporting information

S1 File Heart failure patient survey. (XLSX)
